# IRE1 inhibition perturbs the unfolded protein response in a pancreatic β-cell line expressing mutant proinsulin, but does not sensitize the cells to apoptosis

**DOI:** 10.1186/1471-2121-15-29

**Published:** 2014-07-10

**Authors:** Liling Zhang, Courtney Nosak, Pietro Sollazzo, Tanya Odisho, Allen Volchuk

**Affiliations:** 1 Division of Advanced Diagnostics-Metabolism, Toronto General Research Institute, University Health Network, 101 College Street, TMDT 10-706, Toronto, ON, Canada; 2 Department of Physiology, University of Toronto, Toronto, ON, Canada; 3 Department of Biochemistry, University of Toronto, Toronto, ON, Canada

**Keywords:** Endoplasmic reticulum stress, ER-associated degradation (ERAD), Proinsulin biosynthesis, Pancreatic beta-cells

## Abstract

**Background:**

The Akita mutation (C96Y) in the insulin gene results in early onset diabetes in both humans and mice. Expression of mutant proinsulin (C96Y) causes endoplasmic reticulum (ER) stress in pancreatic β-cells and consequently the cell activates the unfolded protein response (UPR). Since the proinsulin is terminally misfolded ER stress is irremediable and chronic activation of the UPR eventually activates apoptosis in some cells. Here we analyzed the IRE1-dependent activation of genes in response to misfolded proinsulin production in an inducible mutant proinsulin (C96Y) insulinoma cell line.

**Results:**

The IRE1 endoribonuclease inhibitors 4μ8c and MKC-3946 prevented the splicing of the XBP1 mRNA in response to ER stress caused by mutant proinsulin production. Microarray expression analysis and qPCR validation of select genes revealed that maximal upregulation of many UPR genes in response to mutant proinsulin production required IRE1, although most were still increased above control. Interestingly, neither degradation of misfolded proinsulin via ER-associated degradation (ERAD), nor apoptosis induced by prolonged misfolded proinsulin expression were affected by inhibiting IRE1.

**Conclusions:**

Although maximal induction of most UPR genes requires IRE1, inhibition of IRE1 does not affect ERAD of misfolded proinsulin or predispose pancreatic β-cells expressing misfolded proinsulin to chronic ER stress-induced apoptosis.

## Background

Accumulation of unfolded and misfolded proteins in the endoplasmic reticulum (ER) leads to the activation of the unfolded protein response (UPR) that serves to counteract this situation by transiently attenuating protein translation, followed by induction of a transcriptional response that increases the levels of genes involved in ER and secretory pathway function [1]. The UPR is an adaptive program that in metazoans is mediated by three ER stress response sensors, PERK, IRE1 and ATF6. These are ER-localized transmembrane proteins that sense the accumulation of misfolded proteins in the ER and initiate signal transduction cascades that mediate the output of the UPR. The PERK pathway reduces global translation via phosphorylation of eIF2α [2], that in turn enhances translation of the ATF4 transcription factor [3]. IRE1 activation in response to ER stress leads to the splicing of the XBP1 mRNA and translation of the XBP1 transcription factor in mammalian cells [4,5], while ATF6 is an ER-localized protein that is activated by regulated intramembrane proteolysis in the Golgi to release an active transcription factor [6]. Each of these transcription factors regulates genes involved in the UPR, although there is overlap in the genes controlled by these proteins. Furthermore, there is wide variability in the expression and relative abundance of various ER chaperone and co-chaperone proteins in different eukaryotic cells [7], likely due to the nature of the protein products produced by different cell types. Thus, highly specialized cells such as insulin-secreting pancreatic β-cells have a unique chaperone expression profile compared to other cell types and likely have a unique UPR output [7].

In addition to the cell survival output of the UPR, if ER stress remains persistent and these pathways remain active for prolonged periods then apoptosis can be initiated that involves a number of potential pathways, including prolonged expression of pro-apoptotic transcription factors such as CHOP, JNK stress kinase activation, and the IRE1-dependent degradation (or RIDD) activity of IRE1 that non-selectively degrades mRNAs in the vicinity of the ER membrane [8-13].

ER stress has been implicated in contributing to pancreatic β-cell dysfunction and death resulting in the development of diabetes. This is evident in rodents and human patients with certain mutations in the insulin gene that cause misfolding of proinsulin in the ER [14,15] and in rodents and patients with mutations in the PERK gene [16,17]. ER stress has also been implicated in contributing to pancreatic β-cell dysfunction in more common forms of diabetes associated with obesity. Several studies have reported increased ER stress markers in pancreatic islets in rodent models of obesity and diabetes and in humans with type 2 diabetes [18-21]. Furthermore, we recently showed that enhanced chaperone capacity in pancreatic β-cells can improve β-cell function and protect C57Bl/6 mice from developing glucose intolerance in response to a high fat diet [22]. Thus, understanding how pancreatic β-cells respond to ER stress may prove beneficial in developing strategies to improve cell function and survival as potential treatment options for the disease.

To elucidate the UPR in pancreatic β-cells we recently identified gene expression changes resulting from the expression of a mutant proinsulin in an insulinoma cell culture model [23]. Expression of the Akita mutant insulin 2 (C96Y) resulted in induction of various genes involved in ER and secretory pathway function. Furthermore, prolonged expression of the misfolded proinsulin also leads to detection of cell apoptosis in the population [23]. Here we have taken advantage of recently described inhibitors of IRE1 endoribonuclease activity [24,25] to analyse the role of the IRE1/XBP1 pathway in the UPR in this cell line and the effect on ER stress-induced apoptosis. We find that the IRE1 pathway is required for maximal induction of most UPR target genes, but unexpectedly does not sensitize the cells against chronic ER stress-induced apoptosis.

## Methods

### Cell culture

Rat INS-1 insulinoma cells were obtained from Dr. Claus Wollheim (University of Geneva) [26]. INS1 832/13 insulinoma cells were obtained from Dr. Chris Newgard (Duke University) [27]. INS-1 (Insulin 2 C96Y-GFP) cells (clone #4S2) were generated as described [23]. These cell lines were maintained as described in the respective references.

### Microarray analysis

INS-1 (Insulin 2 C96Y-GFP) cells (clone #4S2) were treated with or without Dox (2 μg/ml), Dox with 4μ8c (5 μM), or 4μ8c (5 μM) alone for 48 h. Two independent experiments were performed and total RNA was isolated using TRIzol reagent (Invitrogen) followed by isolation using an RNeasy mini kit (QIAGEN). Assessment of RNA quality and microarray analysis was performed at the University Health Network Microarray Centre as described previously [23].

Genes with multiple probesets were averaged to produce a single fold change value for each gene. Fold change values for both Dox/Untreated and Dox4μ8c/Untreated were log2 transformed. These were then plotted. All analysis was done in R (http://www.r-project.org/).

### RNA isolation and real-time PCR analysis

Total RNA was isolated from rat INS-1 (Insulin 2 C96Y-GFP) cells or mouse islets using TRIzol (Invitrogen) and real-time PCR analysis was performed using the TaqMan Gene Expression system (Life Technologies) as described previously [28]. Gene-specific primers and control β-actin primers were obtained from Life Technologies: Trib3: Rn00595314_m1; HERP: Rn00585371_m1; SDF2L1: Rn01404682_m1; DNAJB9: Rn00562259_m1; GRP78/BiP: Rn01435771_g1; CHOP: Rn00492098_g1; EDEM1: Rn01421307_m1; TXNIP: Rn01533891_g1. The XBP1 splicing assay was performed as described previously [28].

### Cell apoptosis assay

Cell apoptosis was measured using the cell death detection ELISA kit (Roche) according to the instructions provided in the kit and in reference [23]. The ELISA assay detects oligonucleosomes in the cytosol, as an indicator of apoptotic cells.

### MTS cell viability assay

INS-1 (Insulin 2 C96Y-GFP) cells (clone #4S2) cells were either left untreated or treated with 2 μg/ml doxycycline, 2 μg/ml doxycycline and 5 μM 4μ8C or 5 μM 4μ8C alone. After 48 h 50,000 cells/100 μl of media from each treatment well were seeded into a 96-well plate in duplicates. The CellTiter 96 AQ_ueous_ Non-Radioactive Cell Proliferation Assay MTS (Promega, #G5421) was performed according to the instructions provided in the kit. Briefly, 20 μl of the combined PMS/MTS mixture was added to each well and incubated for 4 h at 37°C and 5% CO_2_. The absorbance at 490 nm was then measured with a plate reader.

### Western blot analysis

Proteins were resolved using 10% SDS-PAGE gels or 4-12% NuPAGE gels (Invitrogen) and transferred to nitrocellulose membranes as described in [28]. Antibodies: γ-tubulin, Sigma-Aldrich (T6557); GM130, BD Biosciences (G65120); GFP, Clontech, (632381); KDEL, StressGen, (SPA-827); Insulin, Santa Cruz Biotech. (SC-9168); cleaved caspase 3, Cell Signaling, (9661); Phospho-eIF2α, Cell Signaling, (9721); Herp (provided by Dr. Linda Hendershot, St. Jude Children’s Hospital, Memphis, TN).

## Results

**Figure 1:**
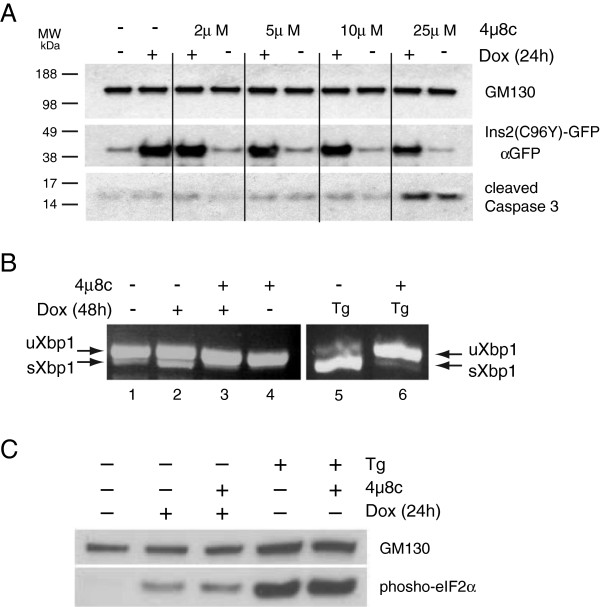
**Effect of IRE1 inhibitor 4μ8c on mutant proinsulin expression, XBP1 splicing and eIF2α phosphorylation. A**. Insulin 2 C96Y-GFP cells were treated or not with 2 μg/ml doxycycline (Dox) for 24 h in the presence or absence of the indicated concentrations of the IRE1 inhibitor 4μ8c. Cell lysates were prepared and immunoblotted for GFP to detect the mutant proinsulin, cleaved caspase 3 and loading control protein GM130. **B**. Insulin 2 C96Y-GFP cells were treated or not with 2 μg/ml Dox for 48 h in the presence or absence of 5 μM 4μ8c and total RNA was isolated. In lanes 5 and 6 the cells were incubated in 1 μM thapsigargin (Tg) for 1 h prior to RNA isolation. Unspliced (u) and spliced (s) XBP1 cDNA were amplified by RT-PCR. Result is representative of 3 independent experiments. **C**. Insulin 2 C96Y-GFP cells were treated as indicated and immunoblotted with phospho-eIF2α and GM130 antibodies.

Expression of a mutant proinsulin C96Y-GFP fusion protein causes ER stress, induction of the UPR and apoptosis in a cultured insulinoma cell line we generated previously [23]. To define the role of the IRE1 pathway in the UPR in this model system we used a recently described IRE1 inhibitor 4μ8c that specifically inhibits the endoribonuclease activity of IRE1 and prevents splicing of the XBP1 mRNA in response to ER stress [24]. We initially tested whether 4μ8c affects doxycycline-induced mutant proinsulin-GFP expression and the effect of the inhibitor on cell survival, apoptosis and XBP1 splicing. The compound 4μ8c had no effect on mutant insulin expression induced by doxycycline or cell viability up to 10 μM (Figure 1A). However, at concentrations >25 μM cell loss was observed and apoptotic cells were detected as monitored by cleaved caspase 3 protein expression (Figure 1A). Consequently, for all subsequent experiments 5 μM 4μ8c was used. At this concentration XBP1 splicing in response to mutant proinsulin expression or thapsigargin treatment was completely prevented (Figure 1B; compare lanes 2 and 3; 5 and 6). As expected, the inhibitor had no effect on mutant proinsulin or thapsigargin-induced activation of the PERK pathway as monitored by Ser51 phosphorylation of eIF2α (Figure 1C).

**Figure 2:**
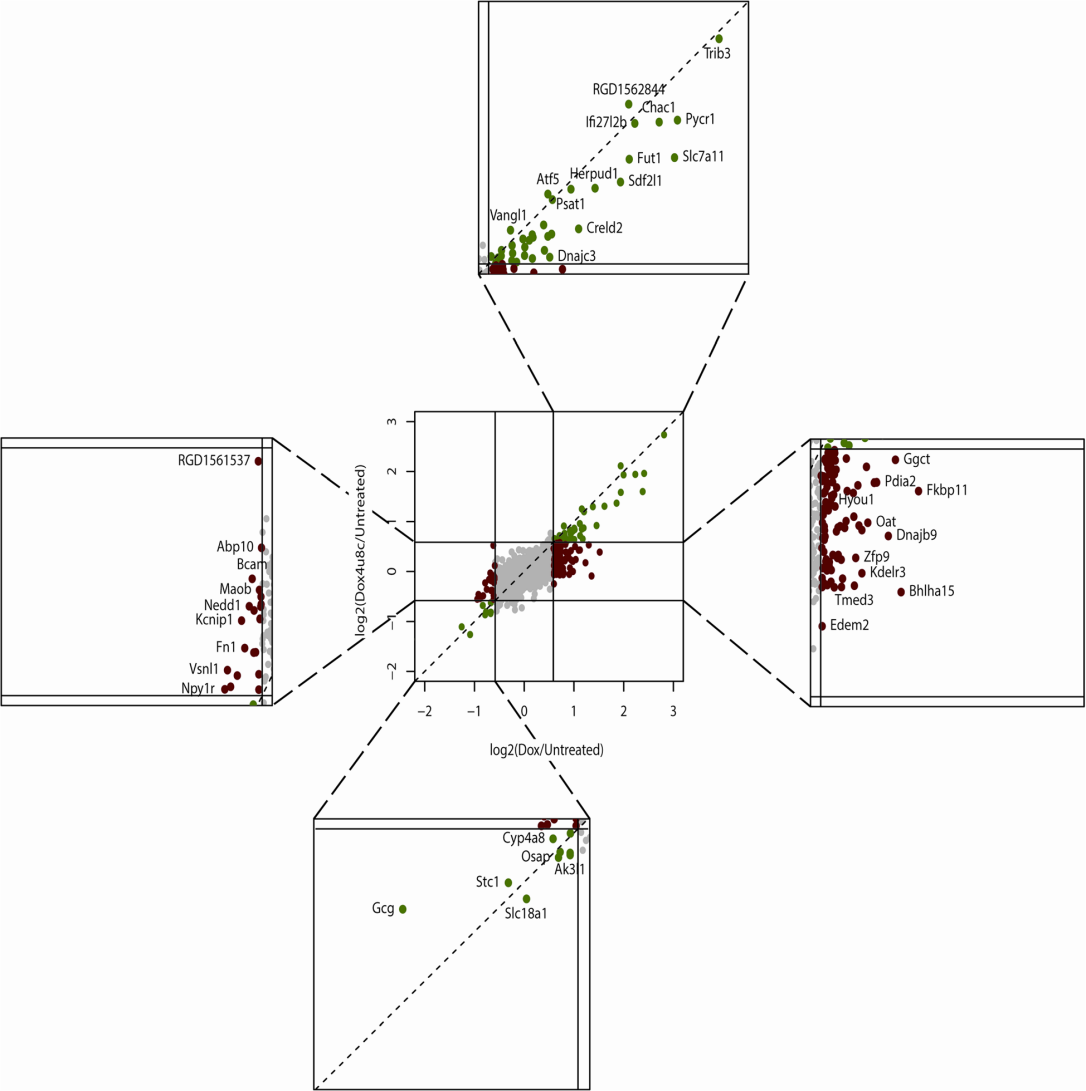
**Effect of IRE1 inhibition on global changes in mRNA expression in response to mutant proinsulin production.** Insulin 2 C96Y-GFP cells were treated with 2 μg/ml doxycycline for 48 h to induce expression of the insulin 2(C96Y)-GFP fusion protein in the presence or absence of 5 μM 4μ8c. Total RNA was isolated from cells and hybridized to Affymetrix microarray chips. Log2 normalized mRNA fold change values relative to untreated cells were plotted. Regions of the plot where gene expression has changed greater than 1.5 fold has been expanded and labeled with selected gene names. Gene expression changes >1.5 fold resulting from doxycycline-induced expression of mutant proinsulin (top and right boxes). Genes no longer induced >1.5 fold in the presence of 4u8c are indicated in red (right box), while those still induced >1.5 fold in the presence of 4u8c are indicated in green (top box). Genes reduced >1.5 fold resulting from doxycycline-induced expression of mutant proinsulin (left and bottom boxes). Genes no longer reduced >1.5 fold in the presence of 4u8c are indicated in red (left box), while those still reduced >1.5 fold in the presence of 4u8c are indicated in green (bottom box). Results are from N=2 independent experiments.

To examine the effect of IRE1 inhibition on global mRNA expression in response to misfolded proinsulin expression, we treated cells with Dox for 48 h to induce mutant proinsulin in either the presence or absence of 4μ8c and performed microarray analysis from two independent experiments. The inhibitor alone did not affect gene expression changes ≥1.5 fold. Doxycycline treatment lead to ≥1.5 fold induction of ~120 genes, most of which were previously observed to be increased by mutant proinsulin expression [23]. This is summarized in Figure 2 and highlighted in the top and right boxes. Surprisingly, a large subset of these genes (~70%) are no longer upregulated ≥1.5 fold (right box, red), while ~30% are still upregulated when the inhibitor was added in the presence of Dox (top box, green). However, genes that were still induced ≥1.5 fold in the presence of the inhibitor usually exhibit lower expression than with Dox alone (Additional file 1: Table S1). The induction of only 6 genes appeared to be not affected by the inhibitor. Thus, it appears that the IRE1 pathway contributes to the induction or maximal induction of the majority of genes in response to mutant proinsulin expression.

**Figure 3:**
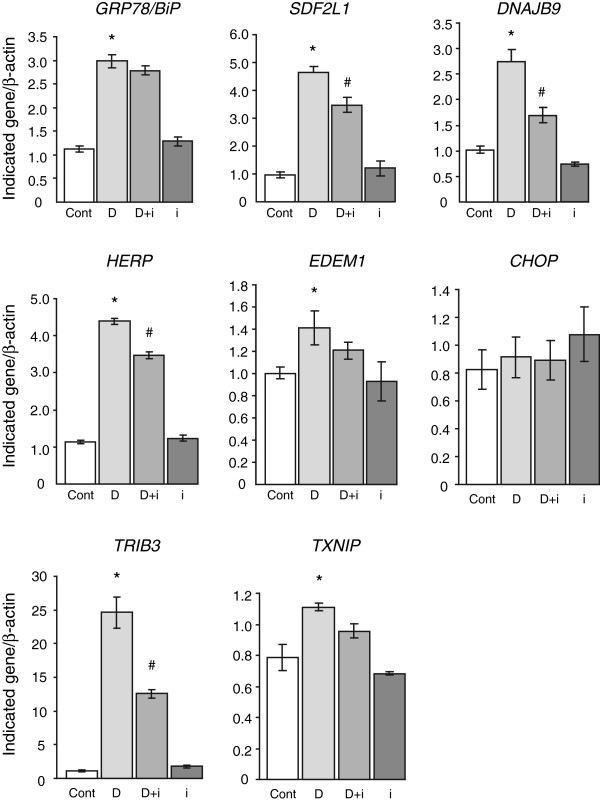
**Validation of select gene expression changes by qPCR.** Insulin 2 C96Y-GFP cells were treated as in Figure 2 and total RNA was prepared. Real-time PCR was used to analyze the levels of the indicated genes relative to cellular β-actin. Results are from N = 3 independent experiments. Statistical significance was assessed by ANOVA followed by Tukey post hoc test. *p < 0.05 D vs. Cont; #p < 0.05 D + i vs. D. (Cont: Control), (D: Doxycycline), (D + i: Doxycycline + 4μ8c inhibitor), (i: 4μ8c inhibitor alone).

Treatment with Dox for 48 h also leads to the down-regulation of a number of genes ≥1.5 fold (Figure 2, bottom and left boxes). The down-regulation of most of these genes is dependent on IRE1 as the presence of the inhibitor reduces or prevents the down-regulation of the majority of these genes (Figure 2 and Additional file 2: Table S2).The microarray analysis suggests that maximal induction of most genes is dependent on IRE1 activity. We validated some of the well-established UPR genes by quantitative PCR. As shown in Figure 3, IRE1 inhibition had no effect on the induction of the major UPR gene GRP78, but did prevent maximal induction of most of the genes examined, including SDF2L1, DNAJB9/ERdj4, HERP and EDEM1. We also examined pro-apototic genes in response to Dox with or without inhibitor. CHOP mRNA levels are not significantly affected by 48 h mutant proinsulin. However, other pro-apoptotic genes such as Trib3 and TxNIP that are induced by mutant proinsulin expression are reduced by the inhibitor (Figure 3). In summary, most well-established UPR genes are still induced when IRE1 activity is completely inhibited, although the general response appears to be blunted compared to control cells.

**Figure 4:**
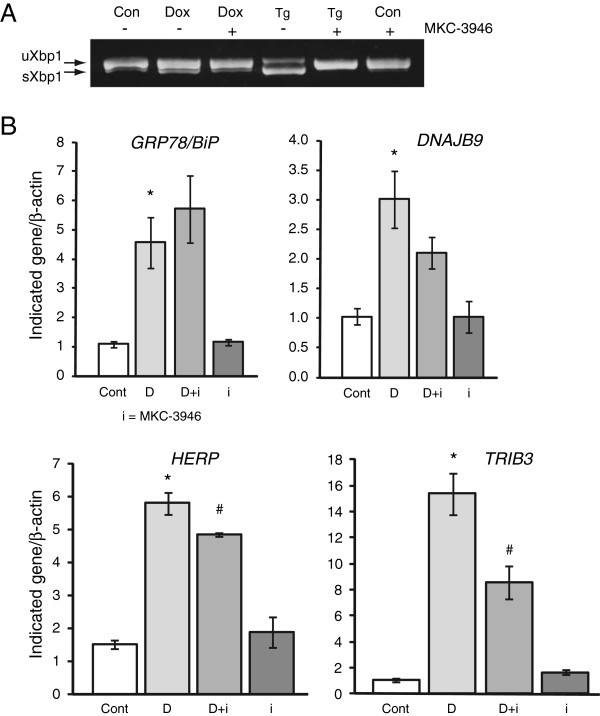
**Effect of MKC-3946 IRE1 inhibitor on XBP1 splicing and UPR gene induction. A**. Insulin 2 C96Y-GFP cells were treated or not with 2 μg/ml Dox for 48 h in the presence or absence of 10 μM MKC-3946 and total RNA was isolated. In lanes 4 and 5 the cells were treated with 1 μM thapsigargin (Tg) for 3 h prior to RNA isolation. **B**. Real-time PCR was used to analyze relative levels of the indicated genes. Results are from N = 3 independent experiments.

We also validated some of these results using a structurally distinct small molecule inhibitor of IRE-1 endoribonuclease activity, MKC-3946 [25]. MKC-3946 also completely inhibited XBP-1 splicing in response to ER stress (Figure 4A) and produced effects on the induction of several UPR genes very similar to 4μ8c (Figure 4B).

**Figure 5:**
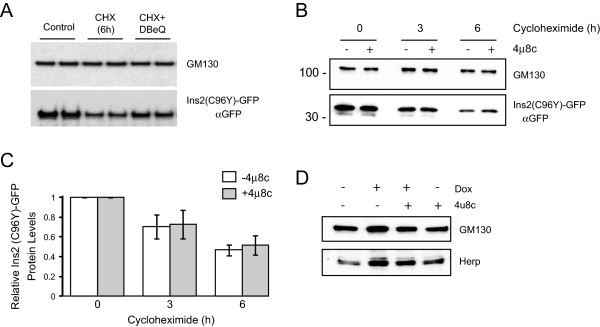
**Effect of IRE1 inhibitor on mutant proinsulin degradation.** Insulin 2 C96Y-GFP cells were treated with Dox for 24 h then treated or not with 100 μM cycloheximide (CHX) with or without p97/VCP inhibitor DBeQ **(A)** or 4μ8c **(B)**. At the times indicated the cells were lysed and proteins were resolved by SDS-PAGE and immunoblotted with anti-GFP and GM130 antibodies. **C**. Western blot results were quantified and normalized to t = 0 h of cycloheximide treatment (Mean ± SE of 4 independent experiments). **D**. Analysis of Herp protein expression by western blot analysis. Result is representative of N = 3 experiments.

**Figure 6:**
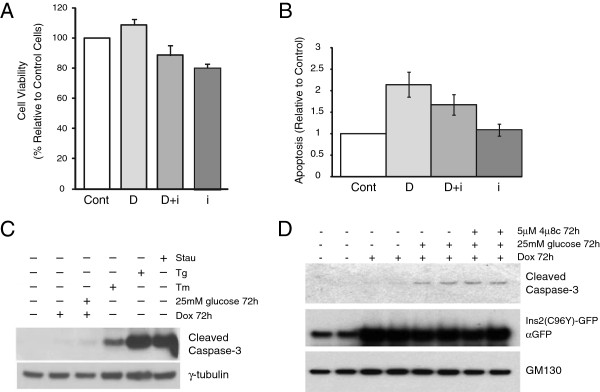
**Effect of IRE1 inhibition on apoptosis induced by misfolded proinsulin expression. A**. Insulin 2 C96Y-GFP cells were treated with or without IRE1 inhibitor 4μ8c (5 μM) in the presence or absence of doxycycline (2 μg/ml). After 48 h the cells were subjected to an MTS assay and % cell viability normalized to control cells. Result is from three independent experiments. (Cont: Control), (D: Doxycycline), (D + i: Doxycycline + 4μ8c inhibitor), (i: 4μ8c inhibitor alone). **B**. Cells were treated as in (A) and apoptotic cells were detected using a Roche Cell Death Detection ELISA assay kit as outlined in the Methods. Results shown represent the mean ± SE of 5 independent experiments. **C**. Insulin 2 C96Y-GFP cells were treated as indicated in the figure and cleaved caspase 3 levels were monitored by western blot analysis. Tg, thapsigargin 1 μM 6 h; Tm, tunicamycin 2 μg/ml 16 h; Stau, staurosporine 1 μM 2 h. **D**. Insulin 2 C96Y-GFP cells were treated as indicated in the figure and cleaved caspase 3 levels were monitored by western blot analysis.

We previously showed that misfolded proinsulin degradation occurs via ER-Associated Degradation (ERAD) [23], a mechanism that retrotranslocates misfolded proteins in the ER lumen to the cytosol for degradation by the proteasome [29]. To further support this notion we examined the effect of inhibiting the ATPase p97/VCP component of the ERAD machinery using the inhibitor DBeQ [30]. Mutant proinsulin was induced by Dox for 24 h, then cycloheximide was added to prevent new protein synthesis and the cells were chased for 6 h with and without DBeQ. Inhibition of p97/VCP reduced mutant proinsulin degradation (Figure 5A). We therefore examined if inhibition of IRE1 activity would affect mutant proinsulin degradation. As shown in Figure 5B,C, the IRE1 inhibitor 4μ8c had no significant effect on misfolded proinsulin degradation. This is consistent with the fact that the ERAD gene Herp is still induced in the presence of IRE1 inhibitors (Figures 3 and 4), as is the Herp protein (Figure 5D). Thus, ERAD degradation of mutant proinsulin is not significantly affected by inhibition of the IRE1 pathway in these cells.Finally, we examined the effect of the IRE1 inhibitor on apoptosis in the mutant insulin expressing cell line. We hypothesized that since activation of the UPR was compromised by the inhibitor that this might sensitize the cells to apoptosis induced by chronic mutant proinsulin expression. General cell viability as monitored by an MTS assay was not significantly affected by mutant insulin expression or the 4μ8c inhibitor (Figure 6A). Mutant proinsulin expression however, induced apoptosis as monitored with a sensitive Cell Death ELISA assay that detects cytoplasmic oligonucleosomes and 4μ8c had no significant effect (Figure 6B). We also monitored cleaved caspase 3 levels by western blot analysis. Cleaved caspase 3 was detected in response to mutant proinsulin expression and was further increased when cells were cultured in the presence of additional stress caused by high glucose (Figure 6C). As expected, the level of cleaved caspase 3 even in the presence of high glucose was much less compared to commonly used thapsigargin or tunicamycin treatments that induce ER stress. The inhibitor had no effect on cleaved caspase 3 levels induced by mutant proinsulin expression in the presence of high glucose (Figure 6D).

## Discussion

In this study we examined the effect of IRE1 pathway inhibition on the UPR in a cell culture model of ER stress caused by expression of a misfolded mutant proinsulin. We found that inhibition of IRE1 endoribonuclease activity using selective inhibitors resulted in a generally blunted gene expression output, although no effect was observed on the kinetics of mutant proinsulin degradation, nor the sensitivity of the cells to apoptosis.

IRE1/XBP-1 has been shown to regulate a variety of genes in various cell types in response to ER stress, mostly related to ER function and the secretory pathway, although the target genes vary depending on the cell type and nature of the stress stimuli [31]. In the proinsulin C96Y-GFP model of ER stress numerous genes related to ER function, the secretory pathway and ER-associated degradation are increased. Here we show that some genes such as GRP78 are completely IRE-1 independent, which is consistent with GRP78 not requiring XBP-1 for its induction [32]. However, most other genes induced require IRE1 at least for maximal induction in response to mutant proinsulin-induced ER stress.

Previously we showed that perturbation of the ERAD pathway either by Herp knock-down or proteasome inhibition significantly perturbs mutant proinsulin degradation and significantly enhances susceptibility to apoptosis [23]. Although the extent of the increase in gene expression was reduced for most genes in the presence of the inhibitor, genes such as those coding for ERAD components are still increased. This may explain the lack of effect of the inhibitor on the degradation of the mutant proinsulin and indicates that IRE1 output is not essential for maintaining ERAD capacity.

Perhaps not surprisingly then, the inhibitor did not increase susceptibility to apoptosis caused by mutant proinsulin expression. Several possibilities could contribute to a lack of effect on cell apoptosis, including reduced RIDD activity in response to chronic stress caused by the misfolded proinsulin, in addition to less induction of some pro-apoptotic genes such as Trb3 [33] and TxNIP [8,9]. Combined with no compromise in ERAD or ability to induce the main ER chaperone BiP/GRP78, cells are no worse off if the IRE1 pathway is inhibited in the context of chronic ER stress caused by mutant proinsulin expression. Our results are consistent with the effect of the inhibitor in other secretory cells where inhibition of IRE1 reduced expansion of secretory capacity, but did not sensitize the cells to ER stress [24].

IRE1 activation results in the production of the XBP1 transcription factor that *in vivo* is required for the development of various secretory cells including pancreatic cells [34-36]. Indeed, disruption of the XBP1 gene in pancreatic β-cells in mice using the RIP-Cre system resulted in hyperglycemia and abnormal β-cell function caused by decreased insulin secretion, decreased insulin granule content and impaired insulin processing [37]. In addition, depletion of XBP1 resulted in constitutive hyperactivation of IRE1 including its RIDD activity [37]. Thus, although inhibition of IRE1 in the context of the Akita insulin mutation does not sensitize the cells to increased apoptosis, it is possible that inhibition of IRE1 *in vivo* in a physiological context might be detrimental to pancreatic β-cell survival.

## Conclusions

In summary, although inhibition of IRE1 compromised the full extent of UPR output in response to chronic ER stress caused by misfolded proinsulin expression, inhibition of IRE1 did not significantly affect ERAD or sensitize the cells to apoptosis. Future studies need to examine the effect of IRE1 inhibition in Akita mice and other more common models of rodent diabetes to determine whether targeting the IRE1 pathway could be of benefit to reducing pancreatic cell death caused by chronic ER stress.

### Availability of supporting data

All supporting data are included as additional files. Microarray data is deposited in the GEO repository, accession number GSE58866.

(http://www.ncbi.nlm.nih.gov/geo/query/acc.cgi?acc=GSE58866).

## Competing interests

The authors declare that they have no competing interests.

## Authors’ contributions

LZ, CN, PS and TO generated experimental data, read and edited the manuscript. PS and AV participated in the design of the study. AV participated in the coordination of the study and wrote the first draft of the manuscript. All authors read and approved the final manuscript.

## Supplementary Material

Additional file 1: Table S1 List of genes induced >1.5 fold by mutant proinsulin expression and mean fold-change induction compared to control cells from N = 2 independent microarray experiments. Column three is the mean fold-change induction of the same genes in the presence of the IRE1 inhibitor 4μ8c. Red: genes whose induction was not affected by 4μ8c; Blue: genes whose fold-induction was reduced by 4μ8c, but whose expression was still >1.5 fold. Green: genes whose induction in response to mutant proinsulin expression was no longer >1.5 fold in the presence of the inhibitor. Click here for file

Additional file 2: Table S2 List of genes reduced by >1.5 fold by mutant proinsulin expression and mean fold-change compared to control cells from N = 2 independent microarray experiments. Column three is the mean fold-change induction of the same genes in the presence of the IRE1 inhibitor 4μ8c. Red: genes whose >1.5 fold reduction was not affected by 4μ8c. Microarray source files are deposited in GEO data repository (GSE58866). Click here for file
